# Neural substrates of verbal repetition deficits in primary progressive aphasia

**DOI:** 10.1093/braincomms/fcab015

**Published:** 2021-02-16

**Authors:** Hilary E Miller, Claire Cordella, Jessica A Collins, Rania Ezzo, Megan Quimby, Daisy Hochberg, Jason A Tourville, Bradford C Dickerson, Frank H Guenther

**Affiliations:** 1 Department of Speech, Language, & Hearing Sciences, Boston University, Boston, MA 02215, USA; 2 Department of Neurology, Frontotemporal Disorders Unit, Massachusetts General Hospital & Harvard Medical School, Charlestown, MA 02129, USA; 3 Department of Radiology, Martinos Center for Biomedical Imaging, Massachusetts General Hospital, Charlestown, MA 02129, USA; 4 Department of Biomedical Engineering, Boston University, Boston, MA 02215, USA; 5 The Picower Institute for Learning and Memory, Massachusetts Institute of Technology, Cambridge, MA 02139, USA

**Keywords:** cortical thickness, GODIVA model, phonological working memory, primary progressive aphasia, repetition

## Abstract

In this cross-sectional study, we examined the relationship between cortical thickness and performance on several verbal repetition tasks in a cohort of patients with primary progressive aphasia in order to test predictions generated by theoretical accounts of phonological working memory that predict phonological content buffers in left posterior inferior frontal sulcus and supramarginal gyrus. Cortical surfaces were reconstructed from magnetic resonance imaging scans from 42 participants diagnosed with primary progressive aphasia. Cortical thickness was measured in a set of anatomical regions spanning the entire cerebral cortex. Correlation analyses were performed between cortical thickness and average score across three phonological working memory-related tasks: the Repetition sub-test from the Western Aphasia Battery, a forward digit span task, and a backward digit span task. Significant correlations were found between average working memory score across tasks and cortical thickness in left supramarginal gyrus and left posterior inferior frontal sulcus, in support of prior theoretical accounts of phonological working memory. Exploratory whole-brain correlation analyses performed for each of the three behavioural tasks individually revealed a distinct set of positively correlated regions for each task. Comparison of cortical thickness measures from different primary progressive aphasia sub-types to cortical thickness in age-matched controls further revealed unique patterns of atrophy in the different subtypes.

## Graphical Abstract

**Graphical Abstract:**
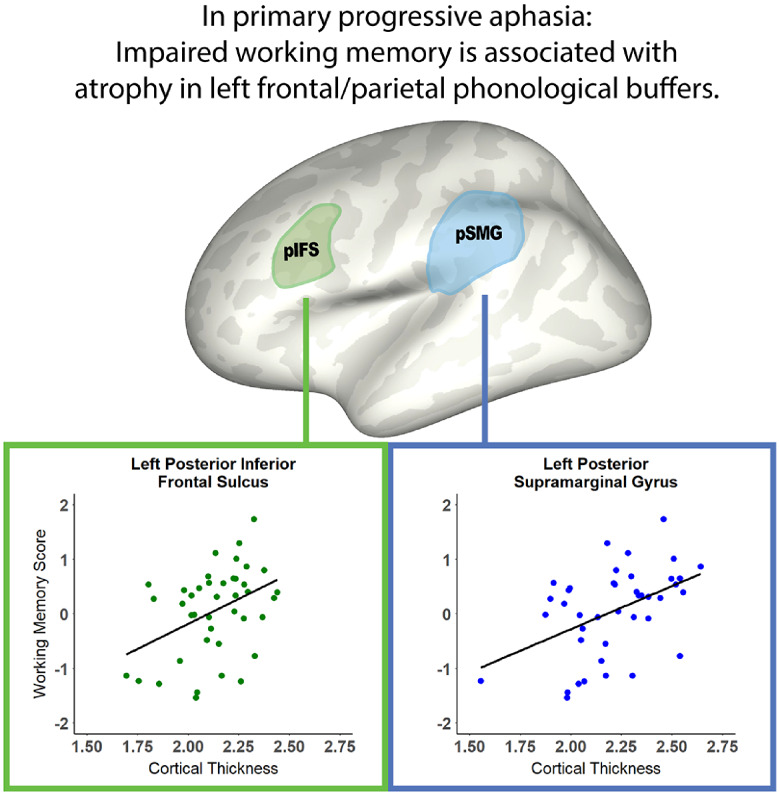

**Abbreviated Summary**

Miller *et al.* identify distinct frontal and parietal verbal working memory buffers by correlating cortical thickness with working memory task performance in primary progressive aphasia patients. Results support proposed phonological buffers in left supramarginal gyrus and inferior frontal sulcus.

## Introduction

Primary progressive aphasia (PPA) is a neurodegenerative syndrome, usually arising from Alzheimer’s disease or Frontotemporal Lobar Degeneration, in which language impairment is the most prominent and initial presenting feature (Mesulam, 2003). Sub-types of PPA further characterize specific patterns of language impairment and expected disease progression (Mesulam *et al.*, 2009; Gorno-Tempini *et al.*, 2011): *semantic-variant PPA (svPPA)* patients demonstrate anomia and impaired single word comprehension; *non-fluent-variant PPA (nfvPPA)* patients demonstrate agrammatism with or without co-occurring apraxia of speech; and *logopenic-variant PPA (lvPPA)* patients demonstrate deficits in lexical retrieval and phonological processing. Cortical thickness measures reveal differential patterns of atrophy across PPA variants (Mesulam *et al.*, 2009, 2012; Rohrer *et al.*, 2010; Sapolsky *et al.*, 2010; Rogalski *et al.*, 2014; Collins *et al.*, 2017), and have been employed to identify neural regions underlying core speech and language domains including articulatory rate, fluency and semantic and syntactic processing (Sapolsky *et al.*, 2010; Rogalski *et al.*, 2011; Mesulam *et al.*, 2015; Cordella *et al.*, 2019). For example, the characteristic anterior temporal atrophy in svPPA is associated with single-word comprehension abilities, whereas distinctive left inferior frontal atrophy in nfvPPA correlates with measures of syntactic processing (Amici *et al.*, 2007; Sapolsky *et al.*, 2010; Rogalski *et al.*, 2011). LvPPA is associated with cortical thinning in the temporoparietal junction, with atrophy here also correlated with the abilities of sentence repetition (Amici *et al.*, 2007; Rogalski *et al.*, 2011; Lukic *et al.*, 2019).

Verbal repetition tasks, such as sentence repetition and digit span tasks, are often used for clinical characterization of the core phonological impairment in lvPPA (Gorno-Tempini *et al.*, 2008, 2011; Foxe *et al.*, 2013; Meyer *et al.*, 2015). However, contradictory evidence suggests that these tasks may not always differentiate lvPPA from other PPA variants or Alzheimer’s disease (Leyton *et al.*, 2014; Beales *et al.*, 2019). Differences in the various verbal repetition tasks used across studies likely contribute to the divergent results. Thus, further investigation of the neural bases of phonological working memory is critical to differentiate underlying neural mechanisms predictive of repetition impairment in PPA patients on common diagnostic tasks.

**Figure 1:**
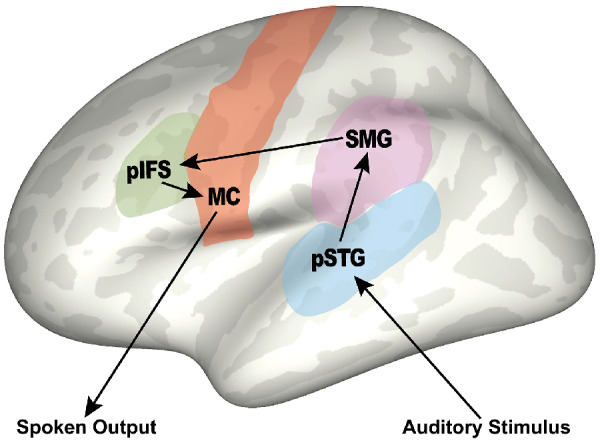
**A simplified account of neural processing in verbal repetition tasks.** Key left hemisphere brain regions involved include: auditory perception in posterior superior temporal gyrus (pSTG), phonological content buffers in supramarginal gyrus (SMG) and posterior inferior frontal sulcus (pIFS), and generation of movement commands in motor cortex (MC), resulting in spoken output of the presented auditory stimulus.

In this study, we focus on working memory-related predictions based on the Gradient Order Directions into Velocities of Articulators (GODIVA) model, which is a neurocomputational model of the processes involved in the planning and sequencing of multisyllabic utterances (Bohland *et al.*, 2010; Guenther, 2016). According to the model, the content of an upcoming utterance is temporarily stored in two distinct sub-regions of prefrontal cortex: a *metrical structure buffer* in bilateral pre-supplementary motor area and a *phonological content buffer* in left posterior inferior frontal sulcus (pIFS; Bohland and Guenther, 2006). The phonological content buffer is responsible for buffering of phonemes in working memory while earlier portions of the utterance are being articulated. We further posit that the phonological content buffer in left pIFS is distinct from a second phonological buffer located in the left supramarginal gyrus that is heavily involved in speech perception and language recognition as shown in Fig. 1.

Previous studies investigating repetition in PPA support the involvement of this temporoparietal phonological buffer in verbal repetition tasks (Amici *et al.*, 2007; Rogalski *et al.*, 2011; Leyton *et al.*, 2012; Lukic *et al.*, 2019). This study seeks to extend this work to test for the involvement of an additional phonological content buffer in left pIFS in sentence repetition and digit span working memory tasks, as predicted by GODIVA model. Successful repetition of sentences or digit sequences during these tasks requires accurate buffering and sequencing of each phoneme for sub-vocal rehearsal and eventual spoken output. The proposed phonological content buffer in left pIFS should therefore be heavily involved in these diagnostic tasks. We also include exploratory whole-brain analyses for each of the three repetition tasks to compare the neural correlates of each task.

## Methods

The study was approved by the Partners Human Research Committee, the Institutional Review Board of Partners HealthCare. All participants provided written informed consent prior to enrolment in the study.

### Diagnostic criteria

Participants included 42 patients with a diagnosis of PPA selected from the PPA Longitudinal Cohort of the Massachusetts General Hospital Frontotemporal Disorders Unit’s Primary Progressive Aphasia Program. For the purposes of this study, PPA participant’s selection criteria were (i) an assessment of repetition and working memory (digit span) performance, (ii) the availability of an MRI scan and (iii) right-handedness. Fifty-one patients from the PPA Longitudinal Cohort were considered for eligibility, with seven patients excluded due to left-handedness and two due to low-quality imaging data. Power calculations indicated that our sample size was adequate to detect a medium strength brain–behavior correlation (*r *=* *0.40) similar to those reported previously in PPA (e.g. Cordella *et al.*, 2019; Petroi *et al.*, 2020), assuming a power of 0.80 and alpha level of 0.05 (one-tailed).

Participants in this cohort undergo a comprehensive clinical evaluation as described previously (Sapolsky *et al.*, 2011, 2014), with diagnosis of PPA and subsequent sub-type classification made by consensus by the neurologist in consultation with the speech–language pathologist. For each participant, we perform an extensive multi-disciplinary assessment including a structured interview of the participant by a neurologist or psychiatrist covering cognition, mood/behavior, sensorimotor function and daily activities; a neurologic examination, including office-based cognitive testing (for cases in this report, BCD); a speech–language assessment performed by a speech–language pathologist (for cases in this report, MQ or DH), including the Progressive Aphasia Severity Scale to specifically assess language impairment from a patient’s premorbid baseline (Sapolsky *et al.*, 2014); an MRI scan with T1- and T2-weighted sequences inspected visually by a neurologist. For each participant, a clinician also performs a structured interview with an informant who knows the participant well, augmented with standard questionnaires. For most of the participants in this report, the protocol included the National Alzheimer’s Coordinating Center Uniform Data Set measures (using version 2.0 previously and currently version 3.0), as well as supplementary measures.

Cases selected for this study had been diagnosed with PPA according to consensus guidelines (Mesulam, 2001; Gorno-Tempini *et al.*, 2011). In accordance with these criteria, all participants exhibited a progressive language impairment with a relative preservation of other cognitive functions. Visual inspection of a clinical MRI ruled out other causes of focal brain damage. No participants harboured a pre-existing psychiatric disorder, other neurological disorder or developmental cognitive disorder. This study included non-fluent-variant PPA patients (nfvPPA; *N* = 13), logopenic-variant PPA patients (lvPPA; *N* = 14) and semantic-variant PPA patients (svPPA; *N* = 15). For 10 out of the 13 patients diagnosed with nfvPPA, both of the two primary inclusion criteria (i.e. apraxia of speech and agrammatism) were met (with two presenting only with agrammatism and one with only apraxia of speech). Detailed speech/language characteristics per diagnostic group are summarized in Table 1.

**Table 1:** Demographic and speech/language characteristics by group, for logopenic variant (lvPPA), non-fluent variant (nfvPPA) and semantic variant (svPPA) patients

	lvPPA ( *n* = 14)	nfvPPA ( *n* = 13)	svPPA ( *n* = 15)
Female, number (%)	8 F (57%)	6 F (46%)	9 F (60%)
Age, y (SD)	71.3 (8.1)	69.4 (8.4)	64.7 (7.3)
Education, y (SD)	16.2 (3.2)	15.8 (3.4)	16.3 (1.9)
Time from Diagnosis,^a^ y (SD)	0.7 (1.1)	1.0 (2.3)	0.9 (1.1)
Mean CDR Language Box Score^b^ (SD)	0.8 (0.4)	0.8 (0.6)	1.1 (0.5)
Mean PASS subdomain scores^b^ (SD)			
Articulation	0.1 (0.3)^c^	1.0 (0.9)^d^^,^^e^	0.0 (0.1)^c^
Fluency	0.5 (0.3)^c^	1.0 (0.6)^d^^,^^e^	0.2 (0.3)^c^
Syntax	0.4 (0.3)	0.7 (0.5)^e^	0.3 (0.3)^c^
Word retrieval	1.0 (0.4)^c^	0.6 (0.2)^d^^,^^e^	1.1 (0.5)^c^
Repetition	0.8 (0.3)^c^^,^^e^	0.4 (0.3)^d^	0.3 (0.3)^d^
Auditory comprehension	0.7 (0.5)	0.4 (0.3)	0.5 (0.5)
Single-word comprehension	0.2 (0.2)^e^	0.0 (0.1)^e^	1.0 (0.5)^d^^,^^c^
Mean PASS score, combined sub-tests	0.5 (0.2)	0.6 (0.3)	0.5 (0.2)
WAB-Repetition score (SD)	71.3 (15.2)^c^^,^^e^	88.8 (14.0)^d^	86.5 (7.6)^d^
Forward Digit Span score (SD)	4.2 (1.6)^e^	5.5 (1.3)	6.7 (1.2)^d^
Backward Digit Span score (SD)	2.5 (1.6)^e^	3.3 (0.9)	4.0 (1.6)^e^

Abbreviations: CDR, Clinical Dementia Rating; PASS, Progressive Aphasia Severity Score; SD, standard deviation, *n*, number of participants diagnosed with each PPA variant.

a Time in years between diagnosis date and initial study visit.

b CDR Language sub-score and all PASS sub-test scores are clinician-rated scores on an interval score where 0 = no impairment; 0.5 = very mild impairment; 1 = mild impairment; 2 = moderate impairment and 3 = severe impairment.

Differences in group means (*post-hoc t*-test, *P* < 0.05) from:

c nfvPPA

d lvPPA

e svPPA.

### Behavioural measures

Participants completed the Repetition subtest of the Western Aphasia Battery-Revised (WAB-R; Kertesz, 2007), which included 15 stimuli items that vary in length. The sub-test includes seven single words (1–3 syllables) and eight phrases/sentences (5–14 syllables). Each word, phrase or sentence was read aloud to the participant and participants were instructed to repeat. Points per item were determined based on the standardized scoring guidelines. Points were deducted for omissions of phonemes, syllables or entire words, as well as for phonemic substitutions and additions. Points were not deducted in the case of a timely self-correction of phonemic error or an intelligible sound distortion (i.e. motor speech impairment was not penalized). Stimuli were eligible for scoring only after the first administration. In this study, the reported total score for the WAB-Repetition subtest refers to the overall percent correct (out of 100 possible points) across all stimuli.

Participants also completed Digit Span Forward and Digit Span Backward sub-tests from the Uniform Data Set (v3.0) neuropsychological test battery (Weintraub *et al.*, 2009). The Digit Span sub-tests each comprise 14 stimuli digit sets varying in span length (3–9 digits for Forward sub-test; 2–8 digits for Backward sub-test). For each span length, there are two stimuli digit sets. Each digit set was read aloud to the participant and the participant was instructed to repeat those numbers in either the exact order they heard them (Digit Span Forward) or to repeat them back in the reverse order (Digit Span Backward). Responses for each digit set were scored as correct/incorrect, and no partial points were awarded. In these sub-tests, patients were not penalized for either phonological or articulatory errors, provided that the response was intelligible. Testing was discontinued after two consecutive failures on the same span length. In this study, the reported total score refers to the length of the longest correctly repeated sequence. If a participant was unable to correctly repeat the shortest length sequence at least once, they received a total score of zero.

### Structural MRI acquisition and analysis

Imaging data for all PPA patients were acquired on a 3-Tesla Siemens Magnetom Tim Trio system at Massachusetts General Hospital, using a 12-channel phased-array head coil. For each patient, a structural image was obtained using a standard T1-weighted 3D MPRAGE sequence that varied slightly across individuals. Nineteen patients were scanned using the following parameters: repetition time (TR) = 2530.00 ms, echo time (TE) = 3.48 ms, flip angle = 7.00°, number of inter-leaved sagittal slices = 176, matrix dimensions = 256 × 256, field-of-view (FOV) = 256 mm, voxel size = 1.00 mm isotropic. Nine patients had the parameters: TR = 2530.00 ms, TE = 1.64 ms, flip angle = 7.00°, number of interleaved sagittal slices = 176, matrix dimensions = 256 × 256, FOV = 256 mm, voxel size = 1.00 mm isotropic. Five patients were scanned with TR = 2300.00 ms, TE = 2.98 ms, flip angle = 9.00°, number of inter-leaves sagittal slices = 160, matrix = 240 × 256, FOV = 256 mm, voxel size = 1.00 mm isotropic; and two patients had identical parameters with the exception of number of inter-leaved sagittal slices = 192. Two patients were scanned with TR = 2530.00 ms, TE = 1.61, flip angle = 7.00°, number of inter-leaved sagittal slices = 208, matrix dimensions = 256 × 256, FOV = 256 mm, voxel size = 1.00 mm isotropic; and one patient had identical parameters with the exception matrix = 280 × 280 FOV = 280 mm and TE = 1.63 ms. Three patients were scanned with TR = 2200.00 ms, TE = 1.54 ms, flip angle = 7.00°, number of interleaved sagittal slices = 144, matrix = 192 × 192, FOV = 230 mm, voxel size = 1.198 mm × 1.198 mm × 1.200 mm. One remaining patient was scanned with the following parameters: TR = 2400.00 ms, TE = 2.22 ms, flip angle = 8.00°, number of inter-leaved sagittal slices = 208, matrix dimensions = 300 × 320, FOV = 256 mm, voxel size = 0.80 mm isotropic.

MRI structural images were also obtained for age-matched control participants who did not exhibit any cognitive impairment (*n* = 25; mean age = 67.4 years, SD = 4.9; 12 female). MRI data for control participants were obtained using the following scan parameters: TR = 2300.00 ms, TE = 2.95 ms, flip angle = 9.00°, number of inter-leaved sagittal slices = 176, matrix dimensions = 256 × 256, FOV = 270 mm, voxel size = 1.1 mm × 1.1 mm × 1.200 mm.

Cortical reconstructions were generated for each participant’s T1-weighted image using FreeSurfer version 6.0 (https://surfer.nmr.mgh.harvard.edu; 19 February 2021, date last accessed, Dale *et al.*, 1999; Fischl *et al.*, 1999; Fischl and Dale, 2000; Salat *et al*., 2004). This method has been shown to be reliable in older adults for both spatial localization and absolute magnitude of measurements across multiple scan sessions for the identification of brain–behavior relationships (Dickerson *et al.*, 2008). Each cortical reconstruction was inspected for accuracy and any errors in the grey/white-matter boundary or pial surface segmentation were manually corrected. Each patient’s reconstructed cortical surface was then parcellated using the SpeechLabel cortical labelling system, which parcellates each hemisphere into 66 anatomically based regions-of-interest (ROIs) for fine-scale sub-division of cortical regions involved in the speech network and is described in greater detail in previous work from our labs (Cai *et al.*, 2014; Cordella *et al.*, 2019). Average cortical thickness within each ROI of the SpeechLabel atlas was calculated for each patient.

To identify ROIs demonstrating significant atrophy for each PPA variant compared to controls, independent-sample one-tailed *t*-tests were conducted for each ROI, using a one-tailed statistical threshold of *P *<* *0.001 with FDR corrections. Separate ANOVA analyses were completed to identify the differences in cortical thickness in the hypothesized phonologic buffer ROIs in left pIFS and left posterior supramarginal gyrus (pSMG) between each PPA variant.

### Experimental design and statistical analysis

A principal component analysis was first performed for the three working memory scores, which revealed that the three working memory measures contributed essentially equally to the first principal component (coeff = 0.56, 0.60, 0.57, respectively, for backward digit span, forward digit span and the WAB-Repetition sub-scores). Therefore, an average working memory score for each subject was obtained by averaging standard *Z*-score values for each of the three tests. This average working memory score followed a normal distribution, per Shapiro–Wilk test. There were no significant effects for age, gender or total brain volume for either of the hypothesized brain regions or for performance on any of the three working memory tasks.

First, our primary hypothesis as to the association between working memory performance and cortical thickness in left pIFS and left pSMG was assessed using one-tailed Pearson bivariate correlation analyses, with a Bonferroni correction applied to account for multiple comparisons, resulting in an α-level of 0.025. A one-tailed analysis was performed due to the unidirectional hypothesis of reduced working memory performance with cortical thinning in these two ROIs.

Next, an exploratory uncorrected whole-brain analysis was performed to identify additional cortical regions that demonstrate a significant relationship with overall working memory performance. Again, one-tailed Pearson bivariate correlations were performed due to the unidirectional hypothesis of cortical thinning associated with reduced working memory performance. Separate whole-brain one-tailed Spearman correlation analyses were also conducted for each working memory task in order to evaluate task differences. All exploratory correlation analyses used an α-level set at 0.05 due to the exploratory nature of the analyses and were performed in IBM SPSS Statistics 25 for Windows.

### Data availability

The analysed data sets are available from the corresponding author on reasonable request.

## Results

### Brain atrophy patterns by clinical sub-type

**Figure 2:**
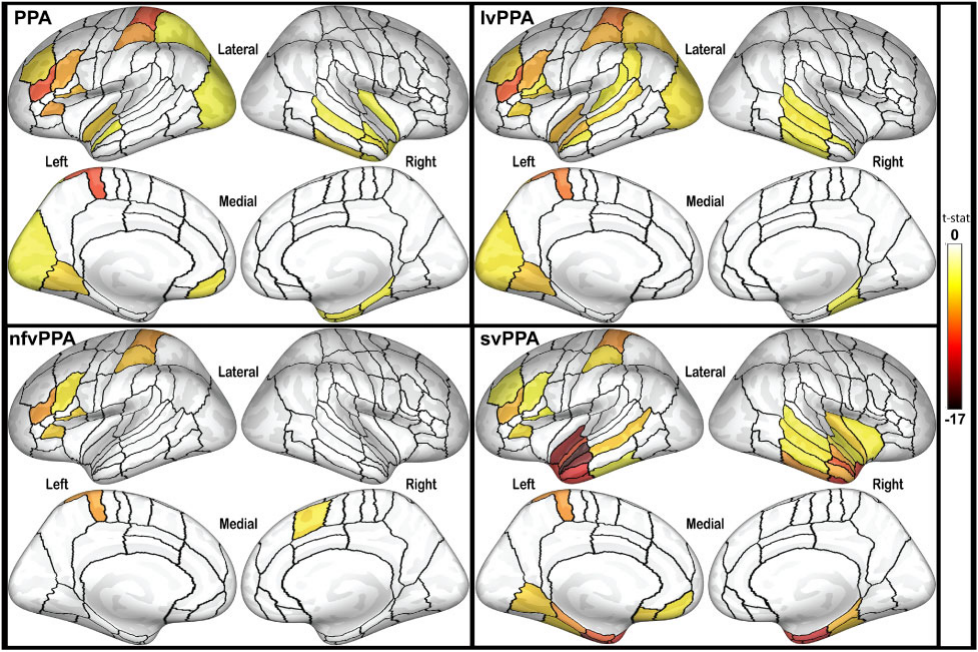
**Atrophy patterns in PPA variants.** Inflated cortical surfaces show ROIs with significantly thinner cortex compared to controls. The colour scale represents *t*-statistic of the effect, with false-discovery rate correction set at 0.001 for each comparison. PPA, primary progressive aphasia, all variants combined; lvPPA, logopenic variant primary progressive aphasia; nfvPPA, non-fluent variant primary progressive aphasia; svPPA, semantic variant primary progressive aphasia.

Cortical thickness measures in this study revealed differential patterns of left hemisphere atrophy across PPA variants (Fig. 2, atrophy maps), largely in line with previously described characteristic atrophy in anterior temporal gyri for svPPA patients, in inferior frontal gyrus for nfvPPA patients and in the temporoparietal junction for lvPPA patients (Rohrer *et al.*, 2010; Sapolsky *et al.*, 2010; Gorno-Tempini *et al.*, 2011; Mesulam *et al.*, 2012; Rogalski *et al.*, 2014). No significant temporal lobe atrophy was observed in the nfvPPA group.

ANOVA analysis revealed a significant group effect in both left pIFS [*F*(2, 39) = 5.55, *P *=* *0.008] and left pSMG [*F*(2,39) = 10.46, *P *<* *0.001]. Bonferroni *post-hoc* comparisons revealed a significant difference in left pIFS cortical thickness only between lvPPA and svPPA patients (*P *=* *0.006; lvPPA: 2.03 ± 0.15; svPPA: 2.23 ± 0.12; nfvPPA: 2.12 ± 0.21). Differences in left pSMG thickness were present between lvPPA and nfvPPA patients (*P *=* *0.002; lvPPA: 2.02 ± 0.05; nfvPPA: 2.30 ± 0.05) and lvPPA and svPPA patients (*P *=* *0.001; svPPA: 2.32 ± 0.05).

### Brain–behavior correlations

**Figure 3:**
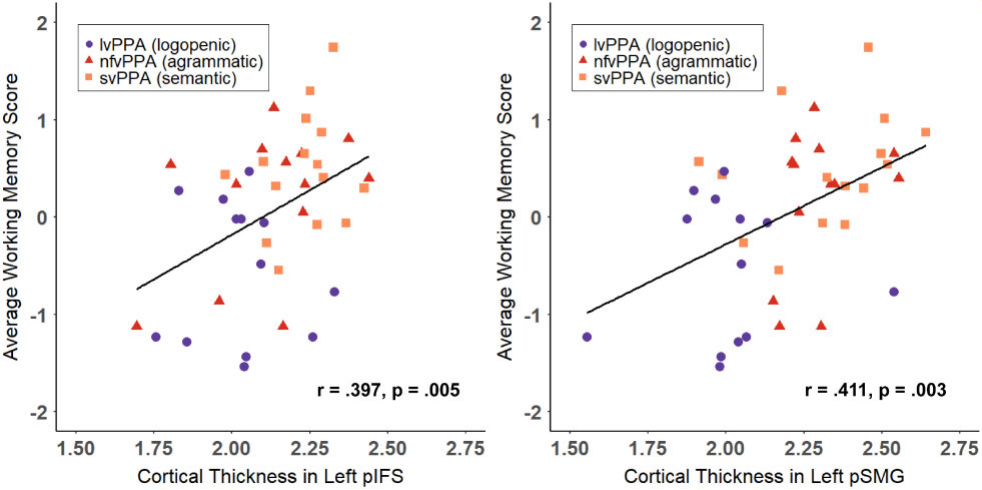
**Scatter plots showing the relationship between average working memory performance and cortical thickness in hypothesized ROIs**. Mean cortical thickness in left posterior inferior frontal sulcus (pIFS, left panel) and left posterior supramarginal gyrus (pSMG, right panel) are plotted as compared to each participant’s average working memory score, obtained from the mean of *Z*-scores from forward digit span, backward digit span and the Western Aphasia Battery-Sentence Repetition sub-scores. Logopenic variant individuals (lvPPA) are shown as purple circles, non-fluent variant individuals (nfvPPA) are shown as red triangles, and semantic variant individuals (svPPA) are shown with orange squares. Solid line shows linear trend for combined group.

Pearson correlations revealed significant correlations between the average working memory score and cortical thickness in left pIFS (*r* = 0.397, 95% CI *r* > 0.155, *P *=* *0.005) and left pSMG (*r* = 0.411, 95% CI *r* > 0.172, *P *=* *0.003). Scatter plots displaying cortical thickness in each of these two regions as compared to average working memory scores for each subject are shown in Fig. 3.

**Figure 4:**
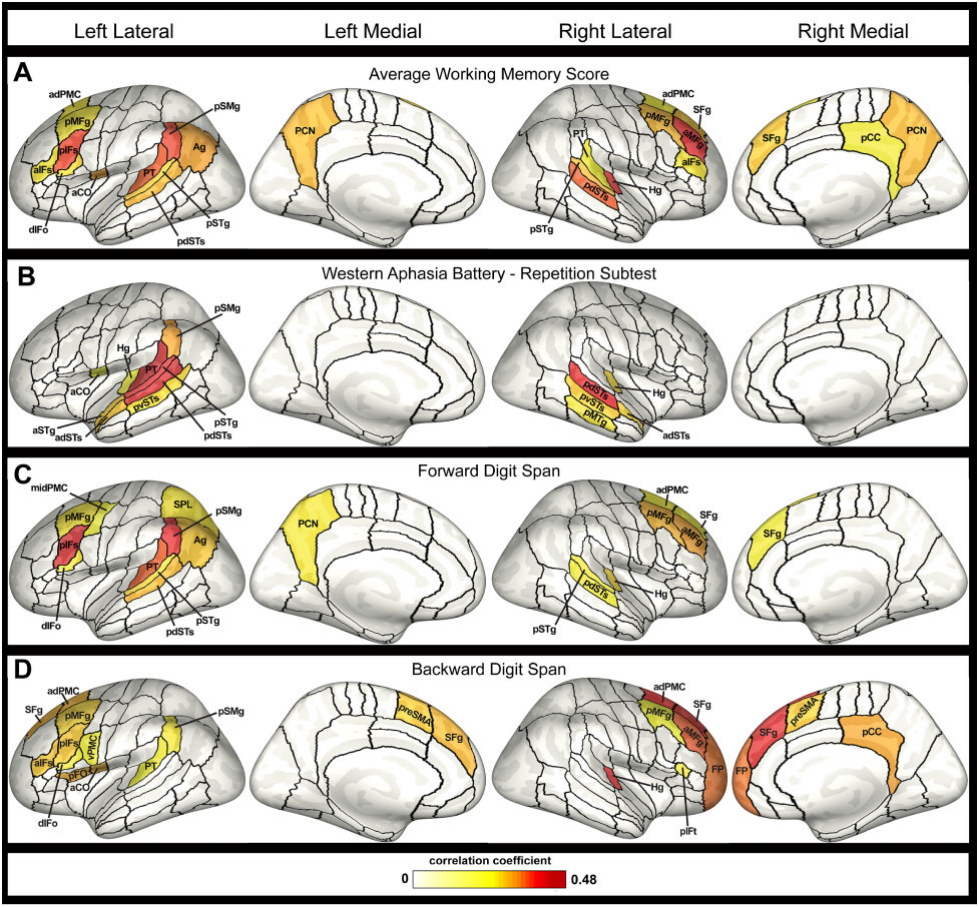
**Correlations between cortical thickness and repetition performance.** Inflated cortical surfaces show ROIs with significant correlations. Medial and lateral surfaces of both left and right hemispheres are shown, with colour map reflecting the strength of the correlation coefficient, thresholded at *P *<* *0.05, uncorrected. (**A**) ROIs correlated with the average working memory score, obtained from an average of *Z*-score values from each of the three individual tests with each of the three repetition tasks; (**B**) ROIs correlated with performance on the Western Aphasia Battery-Repetition sub-test; (**C**) ROIs correlated with forward digit span score; (**D**) ROIs correlated with backward digit span score. Abbreviations: aCO, anterior central operculum; adPMC, anterior dorsal premotor cortex; adSTs, anterior dorsal superior temporal sulcus; Ag, angular gyrus; aIFs, anterior inferior frontal sulcus; aMFg, anterior middle frontal gyrus; aSTg, anterior superior temporal gyrus; dIFo, dorsal inferior frontal gyrus, pars opercularis; FP, frontal pole; Hg, Heschl’s gyrus; midPMC, middle premotor cortex; pCC, posterior cingulate cortex; PCN, pre-cuneus; pdSTs, posterior dorsal superior temporal sulcus; pFO, posterior frontal operculum; pIFs, posterior inferior frontal sulcus; pIFt, posterior inferior frontal gyrus, pars triangularis; pMFg, posterior middle frontal gyrus; pMTg, posterior middle temporal gyrus; preSMA, pre-supplementary motor area; pSMg, posterior supramarginal gyrus; pSTg, posterior superior temporal gyrus; PT, planum temporale; pvSTs, posterior ventral superior temporal sulcus; SFg, superior frontal gyrus; SPL, superior parietal lobule; vPMC, ventral premotor cortex.

Results for exploratory whole-brain correlation analyses with the average working memory score, the WAB-Repetition sub-test score, forward digit span and backward digit span are summarized in Table 2, with significant ROIs shown in Fig. 4. Uncorrected results for the average working memory score revealed correlations with cortical thickness in right Heschl’s gyrus (*r* = 0.415, *P *=* *0.003), right posterior dorsal superior temporal sulcus (*r* = 0.387, *P *=* *0.006), left planum temporale (PT; *r* = 0.385, *P *=* *0.006), right anterior middle frontal gyrus (*r* = 0.412, *P *=* *0.007), in addition to the hypothesized regions. WAB-Repetition scores were most strongly correlated with bilateral posterior dorsal superior temporal sulcus (L: *r*_s_ = 0.461, *P *=* *0.001; R: *r*_s_ = 0.438, *P *=* *0.002), left PT (*r*_s_ = 0.479, *P *=* *0.0007) and left posterior superior temporal gyrus (pSTG; *r*_s_ = 0.452, *P *=* *0.001). WAB-Repetition scores were significantly correlated with cortical thickness in left pSMG (*r*_s_ = 0.341, *P *=* *0.014), but not left pIFS (*r*_s_ = 0.249, *P *=* *0.056). Backward digit span was mostly strongly correlated with right Heschl’s gyrus (*r*_s_ = 0.418, *P *=* *0.003) and right frontal regions including superior frontal gyrus (*r*_s_ = 0.454, *P *=* *0.001), anterior dorsal premotor cortex (*r*_s_ = 0.419, *P *=* *0.003), anterior middle frontal gyrus (*r*_s_ = 0.389, *P *=* *0.005), and frontal pole (*r*_s_ = 0.373, *P *=* *0.007). The two hypothesized ROIs were also correlated with backward digit span (pIFS: *r*_s_ = 0.317, *P *=* *0.02; pSMG: *r*_s_ = 0.282, *P *=* *0.035). The strongest correlations with forward digit span were observed in the two hypothesized phonological buffers (left pIFS: *r*_s_ = 0.458, *P *=* *0.001; left pSMG: *r*_s_ = 0.427, *P *=* *0.002) and left PT (*r*_s_ = 0.388, *P *=* *0.006; for complete results, see Table 2 and Fig. 4). Because of the large number of ROIs tested in this exploratory analysis, none of these correlations survived FDR-correction for multiple comparisons.

**Table 2:** Summary of significant correlations between repetition performance and cortical thickness

ROI	*Average WM*		*WAB-Repetition*		*Forward span*		*Backward span*	
	*r*	*P value*	*r*	*P value*	*r*	*P value*	*r*	*P value*
***Left hemisphere***								
Posterior inferior frontal sulcus	0.397	0.005			0.458	0.001	0.317	0.020
Anterior central operculum	0.343	0.013	0.265	0.045			0.321	0.019
Dorsal IFG, pars opercularis	0.299	0.027			0.28	0.036	0.261	0.047
Posterior middle frontal gyrus	0.284	0.034			0.282	0.035	0.298	0.028
Anterior inferior frontal sulcus	0.287	0.033					0.32	0.019
Anterior dorsal premotor cortex	0.277	0.038					0.327	0.017
Mid premotor cortex					0.259	0.049		
Posterior frontal operculum							0.329	0.017
Superior frontal gyrus							0.325	0.018
Pre-supplementary motor area							0.313	0.022
Ventral premotor cortex							0.274	0.040
Planum temporale	0.385	0.012	0.479	0.0007	0.388	0.006	0.269	0.042
Posterior dorsal STS	0.325	0.018	0.461	0.001	0.330	0.016		
Posterior STG	0.317	0.020	0.452	0.001	0.29	0.031		
Anterior dorsal STS			0.319	0.020				
Anterior STG			0.312	0.022				
Posterior ventral STS			0.300	0.027				
Heschl’s gyrus			0.263	0.046				
Posterior supramarginal gyrus	0.411	0.003	0.341	0.014	0.427	0.002	0.282	0.035
Angular gyrus	0.340	0.014			0.315	0.021		
Pre-cuneus	0.330	0.017						
Superior parietal lobule					0.281	0.036		
***Right hemisphere***								
Anterior middle frontal gyrus	0.412	0.003			0.337	0.014	0.389	0.005
Posterior middle frontal gyrus	0.329	0.017			0.327	0.017	0.271	0.041
Superior frontal gyrus	0.317	0.020			0.267	0.044	0.454	0.001
Anterior dorsal premotor cortex	0.265	0.045			0.271	0.041	0.419	0.003
Frontal pole							0.373	0.007
Pre-supplementary motor area							0.309	0.023
Posterior IFG, pars triangularis							0.261	0.047
Anterior inferior frontal sulcus	0.286	0.033						
Heschl’s gyrus	0.415	0.003	0.303	0.026	0.291	0.031	0.418	0.003
Posterior dorsal STS	0.387	0.006	0.438	0.002	0.261	0.048		
Posterior STG	0.331	0.016			0.262	0.047		
Planum temporale	0.264	0.045						
Posterior ventral STS			0.316	0.021				
Anterior dorsal STS			0.310	0.026				
Posterior middle temporal gyrus			0.282	0.035				
Posterior cingulate gyrus	0.267	0.044					0.344	0.013
Pre-cuneus	0.325	0.018						

ROIs with significant (*P *<* *0.05) correlation coefficients (*r*) and *P-values* with each of the three repetition tasks: Western Aphasia Battery (WAB)-Repetition sub-test; forward digit span; and backward digit span, as well as ROIs significantly correlated with the average working memory (WM) score, obtained from an average of *Z*-score values from each of the three individual tests. IFG, inferior frontal gyrus; ROI, regions-of-interest; STG, superior temporal gyrus; STS, superior temporal sulcus.

## Discussion

We investigated the neural substrates underlying performance on several clinical tests involving phonological working memory (PWM) by examining the relationship between cortical thickness and behavioral performance in patients with PPA. Specifically, we found correlations between average performance across three verbal repetition tasks and cortical thickness in both left pIFS and pSMG in patients with PPA. Our results support the involvement of left pIFS in PWM, as proposed by the GODIVA model of speech sequencing (Bohland *et al.*, 2010) in which left pIFS serves as an output buffer in addition to a separate phonological buffer in temporoparietal cortex. Exploratory whole-brain analyses revealed distinct brain regions that were correlated with scores from each of the three tasks (in addition to some overlap), demonstrating potential differences in the neural correlates underlying successful performance on sentence repetition, forward digit span and backward digit span tasks.

### Evidence of a phonological content buffer in left pIFS

The novel finding of correlations between left pIFS cortical thickness and PWM performance in patients with PPA supports the hypothesized role of left pIFS as a phonological content buffer in the GODIVA model. In this model, left pIFS serves as part of a cortico-basal ganglia-thalamo-cortical planning loop and is specifically responsible for buffering and sequencing the individual phonological units in an upcoming utterance, which are then activated and executed in the correct serial order via projections to ventral premotor cortex for speech output (Bohland *et al.*, 2010; Guenther, 2016). This phonological output buffer should be highly involved in the commonly used tasks analysed in this study. Specifically, impairment of this phonological output buffer should result in poorer verbal repetition performance due to difficulty buffering and sequencing each phoneme prior to motor execution.

Left inferior frontal regions have long been associated with the articulatory rehearsal component of Baddeley’s working memory model (Paulesu *et al.*, 1993; Awh *et al.*, 1996; Baddeley, 2003; Baldo and Dronkers, 2006). Within the GODIVA model, left pIFS (in concert with left ventral premotor cortex) plays a similar role to Baddeley’s articulatory rehearsal process, sequencing through phonological units for either overt speech output or covert rehearsal (Bohland *et al.*, 2010). Bohland and Guenther (2006) demonstrated increased activation in left pIFS during production of more complex syllable strings (e.g. increased number of phonemes per sequence), consistent with the region’s proposed function in which additional neural resources are required to code the serial order of additional phonemes. Moreover, a prior meta-analysis of neuroimaging studies identified left pIFS as the only neural region with preferential activation in verbal (compared to non-verbal) working memory tasks (Rottschy *et al.*, 2012).

Previous studies of verbal repetition in PPA have identified correlations between repetition deficits and atrophy in temporoparietal regions but not prefrontal regions such as pIFS (Amici *et al.*, 2007; Rogalski *et al.*, 2011; Leyton *et al.*, 2012; Lukic *et al.*, 2019). Our study differs from previous work in the use of an ROI-based analysis using the Speech Label parcellation scheme. This parcellation scheme defines subject-specific ROIs to account for inter-subject anatomical variability and allows for finer-scale sub-divisions of critical speech cortical regions, like pIFS, improving the localization of speech and language functions for more sensitive statistical analyses (Nieto-Castañon *et al.*, 2003; Tourville and Guenther, 2012). Discrepant findings are also partly explained by differences in the selected repetition tasks; our individual task analyses demonstrated that left pIFS was not significantly correlated with sentence repetition performance on the WAB, consistent with the previous literature.

### Evidence of a phonological content buffer in left pSMG

Average PWM task performance was also significantly correlated with cortical thickness in left pSMG, consistent with prior theoretical accounts of PWM. This finding replicates recent work, demonstrating correlations between sentence repetition accuracy and cortical thickness in temporoparietal regions, including left SMG, in patients with PPA (Lukic *et al.*, 2019). In patients with lvPPA, atrophy in left SMG is correlated with increased phonologic substitution errors (Petroi *et al.*, 2020) and with impaired naming, presumably due to phonological impairment (Leyton *et al.*, 2012). Similarly, substitution errors and repetition deficits in conduction aphasia have been linked to left SMG damage (Axer *et al.*, 2001; Baldo and Dronkers, 2006; Fridriksson *et al.*, 2010). Left SMG has also been implicated in functional neuroimaging of phonological working memory tasks as a phonological input buffer or the site of Baddeley’s phonological store (Paulesu *et al.*, 1993; Awh *et al.*, 1996; Henson *et al.*, 2000; Bohland and Guenther, 2006; Rottschy *et al.*, 2012; Yue *et al.*, 2019). Notably, our exploratory analysis of individual tasks suggests that this buffer extends from left pSMG into the superior temporal lobe, especially PT. This finding is in line with emergent property models where pSTG and PT act as a sensorimotor interface linking acoustic and phonological representations (Jacquemot and Scott, 2006; Hickok and Poeppel, 2007; Buchsbaum and D’Esposito, 2008; Majerus, 2013).

### Task differences

A distinct set of neural regions (with some overlap) was correlated with each of the three common repetition tasks, with implications for the diagnostic utility of each measure in the clinical management of PPA. This study corroborates previous literature, demonstrating the sensitivity of cortical thickness measures to detect subtle deficits in PPA and identify the neural correlates of numerous speech and language domains (Sapolsky *et al.*, 2010; Rogalski *et al.*, 2011; Cordella *et al.*, 2019). Consistent with Rogalski *et al.* (2011), we found that despite group differences in verbal repetition performance, individual nfvPPA and svPPA patients also presented with subtle repetition impairments (Fig. 3, correlation data), in addition to the more salient deficits in grammar or semantics associated with their primary diagnosis. This study capitalizes on this distribution to analyse the neural substrates of commonly used verbal repetition tasks across PPA variants. Due to the high degree of correlation between the three analysed tasks, there are a number of regions that were correlated with multiple tasks (e.g. ROIs in the temporoparietal junction) that may reflect a common substrate of PWM necessary across tasks. Not surprisingly, some of these ROIs also overlap with the typical atrophy patterns present in lvPPA, consistent with the hallmark repetition deficits in this population.

Forward digit span performance was most strongly correlated with cortical thickness in the hypothesized phonological content buffers in left pSMG and pIFS. This finding suggests that the forward digit span task may be a purer measure of the function of these phonological buffers, requiring less involvement of higher-level language or cognitive systems than other PWM tasks. Significant correlations between task performance and thickness of adjacent temporoparietal ROIs are consistent with the previously reported correlations between left pSTG atrophy and digit span (Leyton *et al.*, 2012). Additionally, bilateral middle frontal gyrus correlations with both digit span tasks likely reflect this region’s suggested role as part of a multi-domain cognitive system (Niendam *et al.*, 2012; Fedorenko *et al.*, 2013). Right middle frontal gyrus may also be involved in number manipulation (Menon *et al.*, 2000; Zago *et al.*, 2008).

The backward digit span task was unique in the contribution of right frontal regions to perform on this task. Bilateral superior frontal gyrus forms part of the frontoparietal control network engaged in sustained attention and executive control (Coull *et al.*, 1996; Vincent *et al.*, 2008; Niendam *et al.*, 2012) with atrophy or lesion shown to impair working memory (Du Boisgueheneuc *et al.*, 2006; Barbey *et al.*, 2013; Nissim *et al.*, 2017). Bilateral pre-supplementary motor area is also considered a core working memory region (e.g. Bohland and Guenther, 2006; Rottschy et al., 2012; Perrachione et al., 2017). Our results support clinical concerns that attentional or executive functioning demands may drive performance on backward digit span tasks more than phonological processing ability (Foxe *et al.*, 2013; Beales *et al.*, 2019); backward digit span performance was less strongly correlated with cortical thickness in presumed phonology regions, instead requiring intact functioning of more general executive function regions.

Performance on the WAB-Repetition subtest was primarily associated with cortical thickness in left temporoparietal junction. This region is associated with repetition deficits in patients with both post-stroke aphasia and PPA (Damasio and Damasio, 1980; Axer *et al.*, 2001; Baldo and Dronkers, 2006; Amici *et al.*, 2007; Fridriksson *et al.*, 2010; Buchsbaum *et al.*, 2011; Rogalski *et al.*, 2011; Lukic *et al.*, 2019), and it is active during PWM tasks in typical speakers (McGettigan *et al.*, 2011; Perrachione *et al.*, 2017; Scott and Perrachione, 2019). The identified neural correlates of the WAB-Repetition task also extend into middle and anterior temporal gyri, which may reflect the semantic and syntactic processing involved in the task (Fedorenko *et al.*, 2010; Friederici and Gierhan, 2013), with lesions here resulting in comprehension deficits and paragrammatism (Sapolsky *et al.*, 2010; Rogalski *et al.*, 2011; Turken and Dronkers, 2011; Matchin *et al.*, 2020). The involvement of these regions suggests that sentence recall may be facilitated by syntactic and semantic knowledge which may outweigh the contribution of the proposed frontal phonological buffer to successful performance on the task (e.g. Baddeley *et al.*, 2009); indeed, the WAB-Repetition task was the only task not significantly correlated with left pIFS. In support of this view, Lukic *et al.* (2019) found that the use of non-meaningful sentences provided increased diagnostic discrimination as it prevented compensatory use of intact semantic processing that may mask phonologic processing deficits in some lvPPA patients.

### Limitations and future directions

In conclusion, the finding of significant correlations between average verbal repetition performance and cortical thickness in both left pIFS and pSMG in a cohort of right-handed PPA patients supports the proposed role of these brain regions. However, we acknowledge several limitations to this work, including the use of only cortical thickness measures in our brain–behavior analyses. White-matter and functional connectivity studies have identified disruptions in the structure and function of key speech and language networks in patients with PPA (Galantucci *et al.*, 2011; Whitwell *et al.*, 2015; Mandelli *et al.*, 2016). A more complete picture may emerge by combining multiple structural and functional measures in a single cohort of patients with PPA.

## Conclusion

Our analyses demonstrate the role of both left pIFS and left pSMG in verbal repetition, but the selected PWM tasks are limited in their ability to sufficiently differentiate deficits in phonological input from output buffer dysfunction, as proposed by prior accounts of PWM. Future work should further isolate and confirm the anatomical correlates of proposed phonological input and output buffers in left pSMG and left pIFS through more precise assessment of deficits in both of these functions. Additionally, our analyses identifying distinct neural correlates of the three repetition tasks are exploratory (i.e. not statistically corrected for the total number of ROIs analysed), allowing for potential Type I errors. These exploratory results suggest significant task differences that should be further investigated in future work to validate these findings.

## Abbreviations

FDR =: false discovery rate
FOV =: field-of-view
GODIVA =: Gradient Order Directions into Velocities of Articulators model
lvPPA =: logopenic-variant primary progressive aphasia
MP-RAGE =: magnetization-prepared rapid gradient-echo
nfvPPA =: nonfluent-variant primary progressive aphasia
pIFS =: posterior inferior frontal sulcus
PPA =: primary progressive aphasia
pSMG =: posterior supramarginal gyrus
pSTG =: posterior superior temporal gyrus
PT =: planum temporale
PWM =: phonological working memory
ROI =: region of interest
svPPA =: semantic-variant primary progressive aphasia
TE =: echo time
TR =: repetition-time
WAB =: Western Aphasia Battery
